# The Role of Cardiac Ganglia in the Prevention of Coronary Atherosclerosis: An Analytical Examination of Cholesterol-fed Rabbits

**DOI:** 10.4274/balkanmedj.galenos.2019.2019.8.97

**Published:** 2020-02-28

**Authors:** Yavuzer Koza, Mehmet Dumlu Aydın, Ednan Bayram, Sare Sipal, Ender Altaş, Celaleddin Soyalp, Enise Armağan Koza

**Affiliations:** 1 Department of Cardiology, Atatürk University School of Medicine, Erzurum, Turkey; 2 Department of Neurosurgery, Atatürk University School of Medicine, Erzurum, Turkey; 3 Department of Pathology, Atatürk University School of Medicine, Erzurum, Turkey; 4 Clinic of Cardiology, Erzurum Training and Research Hospital, Erzurum, Turkey; 5 Department of Anesthesiology, 100. Yıl University School of Medicine, Van, Turkey; 6 Clinic of Anesthesiology, Erzurum Training and Research Hospital, Erzurum, Turkey

**Keywords:** Cardiac ganglia, coronary atherosclerosis, rabbits, vagal nerve

## Abstract

**Background:**

The heart is innervated by the autonomic nervous system, which contributes to the control of the heart’s rhythm and coronary circulation. It has been suggested that the cardiac fibers of the vagus nerve play important roles in controlling circulatory functions and in protecting against atherosclerotic pathologies in coronary arteries.

**Aims:**

To investigate the presence of atherosclerotic differences in the coronary arteries of cholesterol-fed rabbits by measuring the density of cardiac ganglia neurons.

**Study Design:**

Animal experiment.

**Methods:**

This study was conducted using 45 male rabbits. Over a period of 16 weeks, they were kept on an atherogenic diet of water ad libitum and high fat (8.6%) containing saturated fatty acids with 205 mg/kg of cholesterol (1%) per day. Then, their hearts were removed and examined by histopathological methods. Atherosclerotic plaques of the main coronary arteries were examined using the Cavalieri method. Atherosclerosis index values (AIVs) were estimated as the wall surface area/plaque surface area, and the results were analyzed with the Kruskal-Wallis and Mann-Whitney U tests.

**Results:**

While the average atherosclerosis index value was estimated to be ≤8% in 21 animals, the atherosclerosis index value was 9-20% in animals with minor plaque detection (n=11) and ≥20% in animals with major plaque detection (n=10). Increased atherosclerosis index values were more common in animals with low neuron densities than in animals with high neuron densities (p<0.017).

**Conclusion:**

The low neuron density of the cardiac ganglia in cholesterol-fed rabbits is associated with an increased atherosclerotic plaque incidence and volume.

Atherosclerosis is a major cause of coronary artery disease (CAD), and inflammation has a pivotal role in the pathophysiology of atherosclerosis. This association highlights the importance of the inflammatory mediators that are secreted by the vagus nerves (VNs) (1). Acetylcholine (Ach), the principal vagal neurotransmitter, is a potent anti-inflammatory molecule. Moreover, vagal inputs can help to prevent inflammation of the heart (2). Ach diffuses spontaneously over distances of up to many 10s of micrometers and thus reaches effector cells within a comparatively large myocardial area (2,3). Hence, vagal nerve stimulation (VNS) has been found to aid in the prevention of coronary heart disease and cardiac arrhythmias (4).

For many years, it has been thought that no relationship existed between the coronary sclerotic process and the impairment of cardiac vagal activity during ischemic heart disease. A reduction in the activity of the cardiovagal neural network, which is characteristic of ischemic heart disease, and the acute withdrawal of vagal activity that precedes the initiation of ischemia, are not dependent on CAD. Rather, vagal dysfunction is not associated with the impairment of coronary blood flow, conventional atherosclerosis risk factors, and the contractile state of the left-ventricular myocardium (5). It has been also suggested that during ischemic heart disease, reductions in vagal tone are due to impaired hypothalamic parasympathetic control (5). This study was designed to investigate the potential role of the cardiac ganglia in the regulation of normal and atherosclerotic plaque segments using histopathological methods.

## MATERIALS AND METHODS

Forty-five New Zealand male rabbits were used in this study. Ethical approval for this study was given by our institutional research committee, and examinations were performed according to the guidelines set by the ethical committee of our hospital (B.30.2.ATA.0.01.02/2798). All animal experiments complied with the Animal Research: Reporting of *In Vivo* Experiments guidelines and were carried out according to the National Institutes of Health Guide for the Care and Use of Laboratory Animals (NIH Publications no. 8023, revised 1978). All rabbits were housed under standard conditions [a constant temperature  (20-24 °C)], humidity (50-60%), ventilation rate (15 cycles/hour), air supply (with the HEPA filter), and a 12 hours light and dark cycle. Over a period of 16 weeks, they were kept on an atherogenic diet of water ad libitum, high fat (8.6%), and 205 mg/kg cholesterol (1%) with saturated fatty acids per day. After the 16-week feeding period, the animals were given a normal laboratory diet for 4 weeks. Their weights, heart beats, respiration rates, and blood pressure values were recorded. Within 12 weeks, three rabbits died. Anesthesia was induced with isoflurane via a face mask and a subcutaneous injection of 0.2 mL/kg of combination anesthetic (ketamine HCl, 150 mg/1.5 mL, xylazine HCl, 30 mg/1.5 mL, and distilled water, 1 mL); the remaining animals were decapitated. Immediately after intracardiac formalin injection, their hearts were removed, and then fixed in 10% formalin solution for one week. All hearts were examined under an anatomical microscope for gross anatomical properties. For histopathological analysis, 5 µm tissue sections were taken from the venous portion of the cardiac hilum, the epicardial surface of the heart, and the dorso-cranial groove above the interatrial septum, as these areas contain the vast majority of intracardiac ganglia in rabbits. The intrinsic cardiac ganglia were identified using acetylcholinesterase histology (6). Tissue sections were then embedded in paraffin blocks and stained with hematoxylin and eosin. Atherosclerotic plaques in the proximal portion of the main coronary arteries were examined using the Cavalieri volume estimation method (7). Atherosclerosis index values (AIVs) were estimated as the wall surface area/plaque surface area. Cardiac ganglion complexes were examined using stereological methods (8). The neuron stereology and the assessment of CAD were performed by investigators blinded to the experimental condition.

### Statistical analysis

All statistical analyses were performed using a commercially available statistics software package (SPSS® for Windows v. 22.0, Chicago, Illinois, USA). The data are given as mean ± standard deviation. The differences between the AIVs and neuron densities were compared using the Kruskal-Wallis test. When significant differences were found, the Mann-Whitney U test with the Bonferroni correction was used to compare inter-group differences. Differences were considered statistically significant at a Bonferroni-adjusted p value <0.017 (0.05/3).

## RESULTS

The coronary segments of 42 rabbits’ hearts (aged 4±0.5 years old and weighing 3.94±0.45 kg) were serially sectioned and stained histopathologically. The mean heart and respiration rates of rabbits were 281±39/min and 32±8/min, respectively.

### Histopathological findings

Histopathological examinations of heart sections revealed that the atherosclerotic plaques of the coronary arteries in animals with low neuron densities were more significant than the atherosclerotic plaques of coronary arteries in animals with high neuron densities. While the average AIV was estimated to be ≤8% in 21 animals, the average AIV was 9-20% in animals with minor plaque detection (n=11), and ≥20% in animals with major plaque detection (n=10). Increased AIVs were detected more in animals with low neuron densities than in animals with high neuron densities (Table 1).

**Figure 1:**
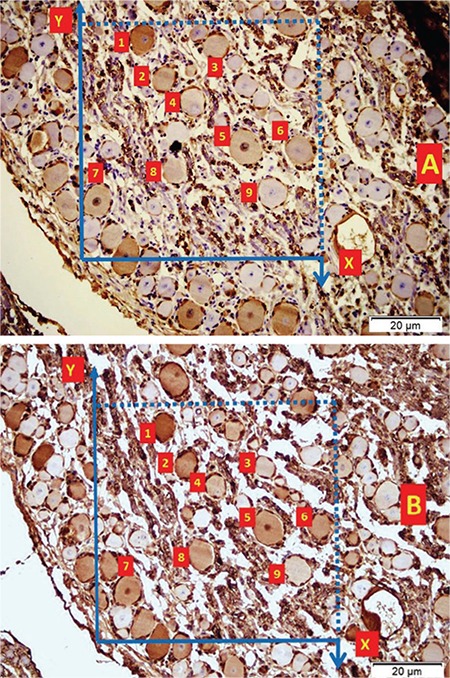
a, b. Stereological cell counting of the cardiac ganglia in a rabbit. Applications of the physical disector method, in which micrographs in the same fields of view (A, B) were taken from two thin, parallel, and adjacent sections that were separated by a distance of 5 μm. The upper and right lines in the unbiased counting frames represent the inclusion lines, and the lower and left lines, including the extensions, are exclusion lines. The neuronal nucleoli touching the inclusion lines were excluded, and the nucleoli profiles touching the inclusion lines inside the frame were counted as disector particles unless their profile extended up to the reference section. The number of neurons from the two disectors occurs in a volume given by the product of the counting frame’s area and the dissectors between the sections. The numerical density of the neurons is calculated as Nv=ΣQ-/txA. Nv is the numerical density of particles, the particle number in a unit volume; ΣQ represents the total number of disector particles; A is the total area of disector sampling; and t is the mean thickness of sections used for the disector counting technique. In this application, the nucleoli that are marked as 1, 2, 4, 7, or 8, 9 are disector particles in Section A, and Section B shows that they are disappeared. The nucleoli that are marked as 3, 5, and 6 are not dissector particles in Section A, and Section B shows that they are disappeared (LM, NSE, ×20).

**Figure 2:**
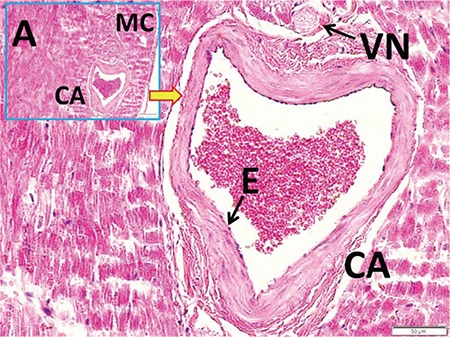
A normal coronary artery in myocardial tissue (LM, H&E, 20/A). A magnified form of the vagal nerve and the endothelium and smooth muscles under the endothelial tissue presented at the base (LM, H&E, ×50). CA: coronary artery, E: endothelium, MC: myocardial tissue, VN: vagal nerve

**Figure 3:**
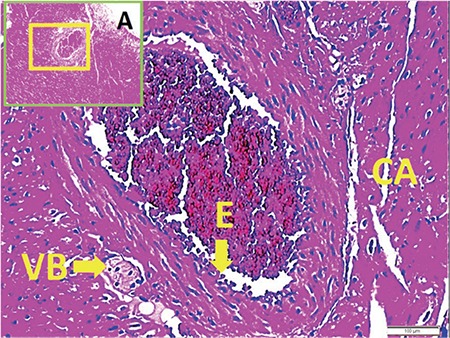
A partially congested coronary artery in the upper left corner of myocardial tissue (LM, H&E, 20/A). At the base, a magnified form of the endothelium and its smooth muscles under the endothelial tissue. The vagal branch is illustrated, as well (LM, H&E, ×100). CA: coronary artery, E: endothelium, VN: vagal nerve

**Figure 4:**
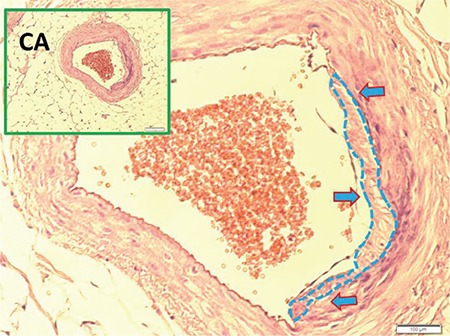
A magnified form of degenerated endothelium (arrows) and its smooth muscles under the endothelial tissue. CA: coronary artery

**Figure 5:**
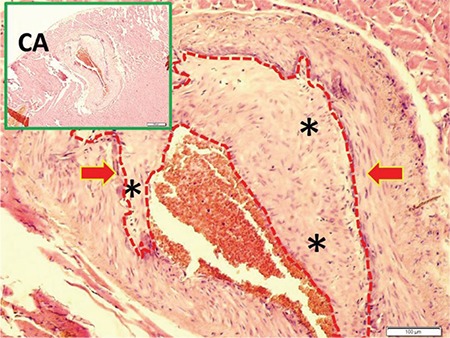
A high degree of atherosclerosis in a coronary artery in the upper left corner of myocardial tissue (LM, H&E, 20). At the base, an atherosclerotic plaque is shown with yellow-red arrows and asterisks (LM, H&E, ×100). CA: coronary artery

Figures 1A and 1B illustrate the stereological cell counting of cardiac ganglia in a rabbit. Figure 2 shows the VN and a normal coronary artery (CA) in its magnified form with the endothelium and smooth muscles under the endothelial tissue. The animal’s average neuron density is 9300±850 mm^3^. Figure 3 reveals a partially congested CA and a vagal branch. Figure 4 depicts a magnified form of a degenerated endothelium and its smooth muscles beneath the endothelial tissue in an animal with an AIV of 11%, and a neuron density of 7800±750 mm^3^. Finally, Figure 5 shows a high degree atherosclerotic CA in the myocardial tissue of an animal with an AIV of 40%, and an average neuron density of 6500±630 mm^3^.

## DISCUSSION

The present study demonstrates that a low neuron density of cardiac ganglia in cholesterol-fed rabbits is associated with an increased incidence and volume of atherosclerotic plaque.

A growing number of anatomical and physiological studies have confirmed that the VN directly affects the right and left ventricles independently of the sinus and atrioventricular nodes (9,10). The VN further gives off superior and inferior cardiac branches, until finally merging with the postganglionic sympathetic neurons to form a complex set of epicardiac ganglionated plexi (11,12).

The central part of the cardiac nervous system forms a complex neural network consisting of ganglionated plexi and interconnecting ganglia and axons (13). Vagal fibers are found both in the perivascular connective tissue and in the adventitia of the arteries, contributing to CA dilation. Unlike sympathetic innervation, which must first synapse within the chain ganglia to innervate the heart with postsynaptic fibers, the parasympathetic fibers synapse at the ganglia located directly on the heart, from which postsynaptic fibers then innervate the target organ (14).

Cardiac ganglia consist of various neuronal components that include parasympathetic, sympathetic, afferent, and interconnecting neurons (15). In humans, every intracardiac ganglion is composed of 200 to 1000 intracardiac neurons, so that each acts as a major local integration center for the intracardiac nervous system (16,17).

Increased sympathetic activity and reduced vagal activity are associated with increased mortality both after myocardial infarction and in heart failure (2,3,4,5,6) and further vagal withdrawal has been documented to precede acute decompensation.

Intracardiac neurons are primarily cholinergic, releasing Ach as their main neurotransmitter and have an inhibitory role in cardiac regulation. Horackova et al. (18) revealed that while most (~75-100%) intracardiac neurons were choline acetyltransferase-positive, only about 10% were tyrosine-hydroxylase-positive. The production of ach in ischemic myocardial areas increases approximately 20 times. Concurrently, afferent vagal fibers trigger a reflex withdrawal of norepinephrine release, which is also increased by ischemia (19,20).

Although increased sympathetic activity and decreased vagal activity are associated with increased mortality, both after myocardial infarction and during heart failure, the precise mechanism is unknown (11,12). Several studies have demonstrated that different cardiac diseases can lead to pathological changes in intrinsic cardiac neurons. Hopkins et al. (21) indicated that in ischemic human hearts, 1/3 of intracardiac neurons display cytoplasmic inclusions, a severe enlargement (66×54 μm vs 40×34 μm for normal neurons), and degenerative changes in their dendrites and axons. Similarly, our results suggest that a decreased neural density can contribute to the development of CAD.

In a previous study that measured the extent of reduced cardiac vagal tone with heart rate variability (HRV) among people with no perceptible signs of arteriosclerosis, significant correlations between diminished activity and myocardial infarction, sudden cardiac death, and coronary bypass surgery were determined (22). In that study, the follow-up period was short (three years), and the source population was relatively young with a small incidence of coronary heart disease. It should also be noted that the rate of fatty streaks in human beings has previously been found to increase from 5% in adolescents (16-20 years of age) to 17% in adults (41-45 years of age), proposing the sustained formation of atherosclerotic lesions (23). Furthermore, in Manfrini et al. (24) study of autonomic nervous system activity, using HRV in 42 patients with single CAD who underwent percutaneous coronary intervention, they determined that the vessel wall that stretched outward behind the plaque was associated with autonomic insufficiency, primarily due to decreased vagal tone. Regardless, it is presently unclear if remodeling can lead to cardiac vagal or if vagal withdrawal can contribute to arterial remodeling. Although the VNS and dilated cardiac microcirculatory vessels have been associated with ameliorated left ventricular contractile dysfunction in patients with severe CAD (25), it remains unclear if the effects of the VNS are being produced by an increased vagal or reduced sympathetic activity. By comparison, several studies have reported that a decrease in cardiac vagal activity during ischemic heart disease is in no way related to coronary atherosclerosis (26,27,28). All the same, the conclusion of these studies was based entirely on HRV.

In a prior study, intravenous (iVNS) therapy was used before coronary reperfusion. It significantly reduced infarct size and preserved cardiac function for an entire month after acute myocardial infarction (29). Although the benefits of iVNS therapy were attributed to the VN’s impact on bradycardia, the VN’s relationship with antiatherosclerosis could play a significant role in animals with a high VN density. This functional study included a canine model of myocardial infarction. In another study, epicardial ganglionated plexus stimulation decreased postoperative inflammatory responses in people (30).

The current study reveals that a decreased neural density of the cardiac ganglia may be associated with the progression and composition of coronary atherosclerosis, in addition to other previously known low-grade systemic inflammatory conditions, such as obesity, hypertension, and metabolic syndrome. Therefore, people with decreased vagal activity, or who lack certain heart-to-brain communication signals, may be especially prone to the development of coronary atherosclerosis. Decreased cardiac vagal activity can initiate or exacerbate CAD. In addition, intracardiac neurons that have been perfused by a diseased CA can undergo pathological alterations that compromise their functions. Sedentary lifestyles, the various stressors of modern life, and other harmful behaviors can lead to the chronic withdrawal of vagal activity. Therefore, potential targets in the treatment of coronary atherosclerosis should include intracardiac neurons. Direct vagal stimulation could also open the door to unique treatment options in the future, such as non-pharmacological antiatherosclerotic treatment strategies for CAD.

To date, several animal models have been conducted for the study of atherosclerosis. Previous rabbit models have primarily used the high cholesterol diet, arterial wall balloon injury, or a combination of these methods for the induction and development of atherosclerosis. However, the effects of the cholesterol diet, feeding period, and balloon injury have yet to be standardized. In addition, long-term feeding can lead to massive inflammatory responses that do not resemble the chronic low-grade inflammatory responses that are associated with human atherosclerosis. It is also important to note that in most atherosclerosis models, animals do not develop human complications, such as plaque rupture, myocardial infarction, stroke, or sudden death.

For full organ preparations and advanced pathophysiological studies *in vivo*, the contractile function of the heart is a challenging caveat, as using *in vitro* specimens on intrinsic cardiac neurons can severely limit interpretation of the relevant findings. In the current study, we were unable to determine if certain plaques were histologically vulnerable to rupture, and we did not measure serum cholesterol or triglyceride levels. The plaque types were neither classified nor assessed immunohistologically. It should also be remembered that the results from the rabbit models are unable to explain the presence of atherosclerosis in humans perfectly. All the same, these models could prove useful during assessments and comparisons of efficacy for new pharmacological interventions.

## Figures and Tables

**Table 1:**
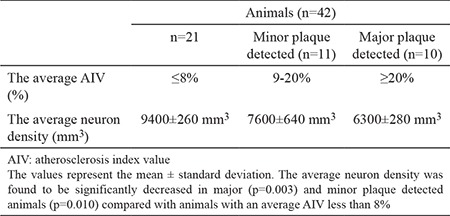
Numerical values of the experiments
